# New Pathogenic Variant in the GLI3 Gene in the First Colombian Patient Associated With Pallister‐Hall Syndrome: A Clinical Report

**DOI:** 10.1002/mgg3.70146

**Published:** 2025-10-09

**Authors:** Sebastián Bonilla‐Navarrete, Luis Eduardo Prieto, Laura Valentina Carvajal, Jorge Andrés Olave‐Rodríguez, Lisa Ximena Rodriguez‐Rojas, Jose Antonio Nastasi‐Catanese

**Affiliations:** ^1^ Fundación Valle del Lili, Departamento de Genética Clínica Cali Colombia; ^2^ Facultad de Ciencia de la Salud Universidad Icesi Cali Colombia; ^3^ Fundación Valle del Lili, Centro de Investigaciones Clínicas Cali Colombia

**Keywords:** genotype–phenotype correlation, hamartoma, novel genetic variant, polydactyly

## Abstract

**Background:**

Pallister‐Hall syndrome (PHS) is an extremely rare genetic disorder. It presents as a polymalformative syndrome affecting craniofacial structures, the central nervous system, limbs, various internal organs, and the endocrine system. It is considered a ciliopathy, as it is associated with loss‐of‐function variants in the GLI3 gene, a transcription factor essential for primary cilium signaling. The syndrome shows marked clinical heterogeneity depending on the type of genetic variant, which makes diagnosis challenging. It is usually suspected at an early age when a hypothalamic hamartoma is associated with polydactyly. Endocrine manifestations are often linked to the hamartoma, further complicating diagnosis.

**Case Presentation:**

A 23‐year‐old Colombian patient presented with a history of hypothalamic hamartoma, gelastic seizures, postaxial polydactyly of hands and feet, and craniofacial dysmorphisms. Physical examination revealed dolichocephaly, bilateral epicanthus, broad nasal bridge, short and broad neck, mild cervical kyphosis, mild scoliosis, micrognathia, bilateral ulnar deviation of the fourth and fifth fingers, and overlapping toes on both feet. No genital anomalies were found. Neuropsychological evaluation reported a borderline intellectual quotient of 78. Whole‐exome sequencing identified a de novo heterozygous pathogenic variant in GLI3 (c.2151del; p.(Gln71HisfsTer16)), confirmed by Sanger sequencing.

**Conclusions:**

We report the first case described in Colombia of a previously unreported truncating genetic variant. We performed a clinical–molecular correlation in a 23‐year‐old adult whose diagnosis of PHS was delayed until adulthood, years after the initial identification of a hypothalamic hamartoma, refractory gelastic seizures, polydactyly, and mild cognitive impairment. This case broadens the clinical spectrum regarding the viability of patients with PHS into adulthood, showing that it is not restricted to the severe neonatal or infantile presentations classically described.

## Graphical abstract

Pallister‐Hall syndrome: from phenotype to molecular diagnosis.

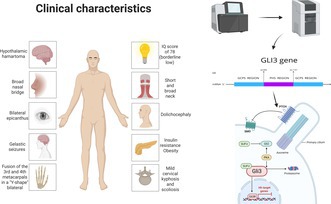

## 1 Introduction

The *GLI3* gene is located on chromosome 7p14.1 and encodes a zinc finger transcription factor that acts as a mediator in the Sonic Hedgehog (SHH) signaling pathway, dependent on primary cilia. This protein is expressed in multiple tissues, primarily in the limbs, brain (with a predominant role in the development of the forebrain or prosencephalon), heart, and kidneys, making it crucial during embryonic development. Consequently, certain malformations, such as hypothalamic hamartoma, pituitary dysplasia, midline defects like holoprosencephaly, craniofacial abnormalities, limb malformations such as polydactyly and syndactyly, renal/adrenal malformations, and congenital heart defects, have been frequently associated with pathogenic or likely pathogenic variants in the *GLI3* gene, either at the somatic or germline level. These phenotypic features resemble a broader group of disorders known as ciliopathies (Motoyama 2006). Depending on the affected region of the *GLI3* gene, various clinical entities can be observed within the clinical spectrum, including postaxial polydactyly type A, polysyndactyly, acrocallosal syndrome, Greig cephalopolysyndactyly syndrome (GCPS), and Pallister‐Hall syndrome (PHS), with the latter two being the only syndromic phenotypes associated with a more severe clinical presentation (McDonald‐McGinn et al. 2010). Clinico‐molecular correlations have become crucial in medical practice, and in the context of the *GLI3* gene, they are essential for distinguishing phenotypes defined by alterations in specific regions of the gene (Kang et al. 1997).

PHS was first described in 1980 as a disease primarily suspected based on the presence of central nervous system abnormalities, most notably hypothalamic hamartoma. This abnormality is often associated with gelastic seizures, which are refractory to treatment during childhood but tend to improve in adulthood. Additionally, callosal dysplasia, intellectual disability, and behavioral disorders may also be present (Hall et al. 1980).

In association with the syndrome, various anomalies can be observed, including limb malformations such as typically postaxial or mesoaxial polydactyly and syndactyly; renal/adrenal abnormalities that can result in fatal outcomes such as adrenal aplasia and adrenal crisis; pulmonary segmentation anomalies; and other endocrinological defects like panhypopituitarism. Cardiac defects, primarily septal and valvular, genitourinary malformations in males, imperforate anus, and symptomatic bifid epiglottis are also noted, among others. PHS follows an autosomal dominant inheritance pattern, with most cases being de novo, which are usually more severe than those with a family history of the disease. These cases are often associated with pathogenic truncating variants, primarily located in the middle third of the *GLI3* gene (Yang et al. 2021).

The prevalence of this condition is not well established due to the few reported cases, but it is estimated to be less than 1 in 1,000,000.

We present a novel pathogenic variant in the *GLI3* gene associated with PHS in a patient from southwestern Colombia, treated at a high‐complexity institution. This variant has not been previously reported in any global genetic variant databases or curated variant collections. Our group has submitted this variant to ClinVar, classifying it as disease‐causing with clear clinical congruence.

## 2 Case Presentation

A 23‐year‐old Colombian male patient, product of a third pregnancy of non‐consanguineous parents, was born via cesarean section at 24 weeks of gestation due to intrauterine growth restriction (IUGR). He required hospitalization for 2 months due to prematurity and associated complications at birth. The patient presented postaxial polydactyly of both hands and the right foot, necessitating multiple surgical interventions from the age of 1 year. Additionally, he had structural focal epilepsy, with episodes of crying/laughing consistent with gelastic/dacrystic seizures, and refractory complex focal seizures. Currently under anticonvulsant treatment with levetiracetam, clobazam, and lacosamide, he exhibited developmental delay from infancy, achieving head control and babbling at 5 months of age, rolling over at 10 months, crawling after 1 year, standing with support at one and a half years, producing bisyllabic words, and walking without support at 2 years of age. He also experienced growth retardation and insulin resistance, which improved with age.

**FIGURE 1:**
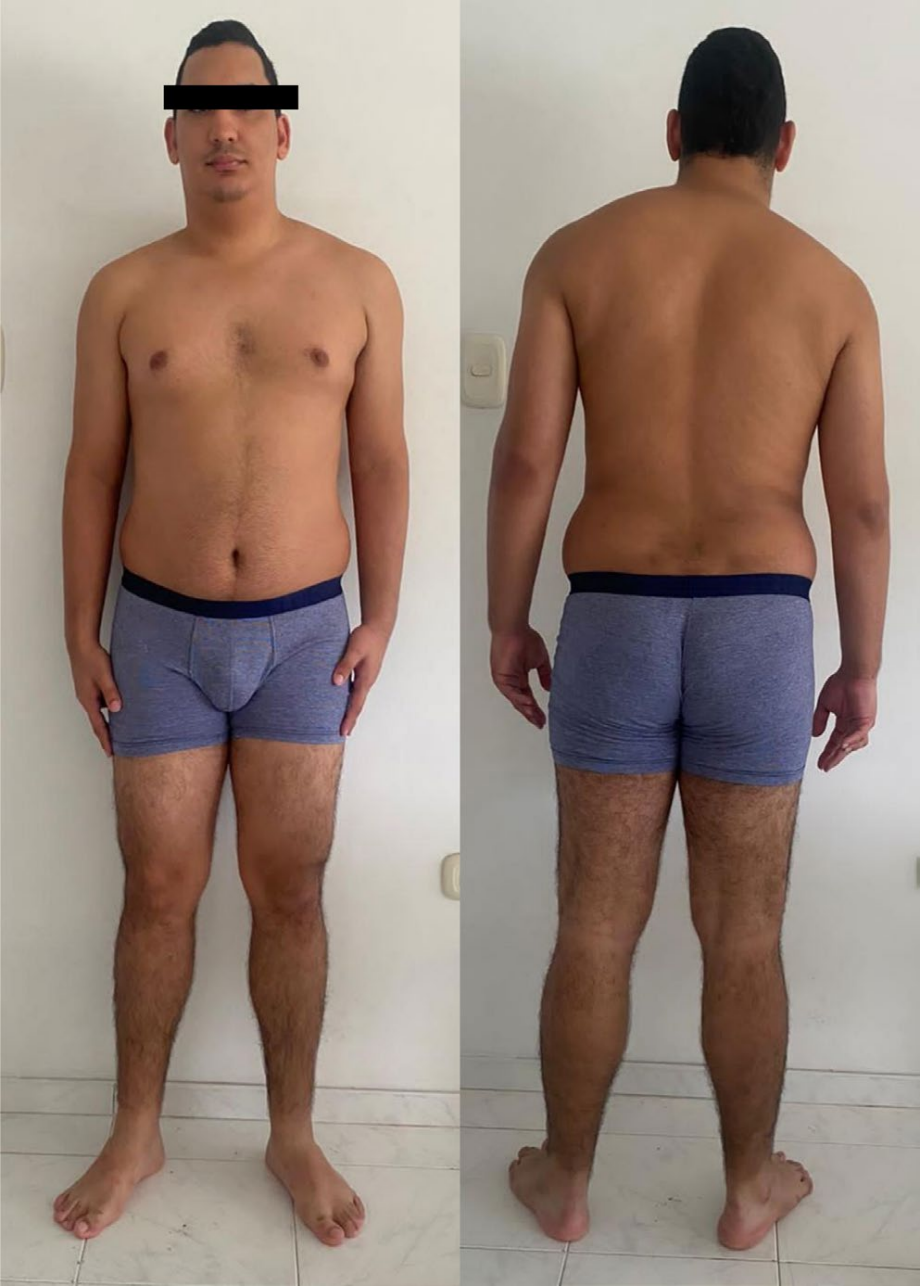
Phenotypic features: Dolichocephaly, broad nasal bridge, mild cervical kyphosis, and mild scoliosis.

**FIGURE 2:**
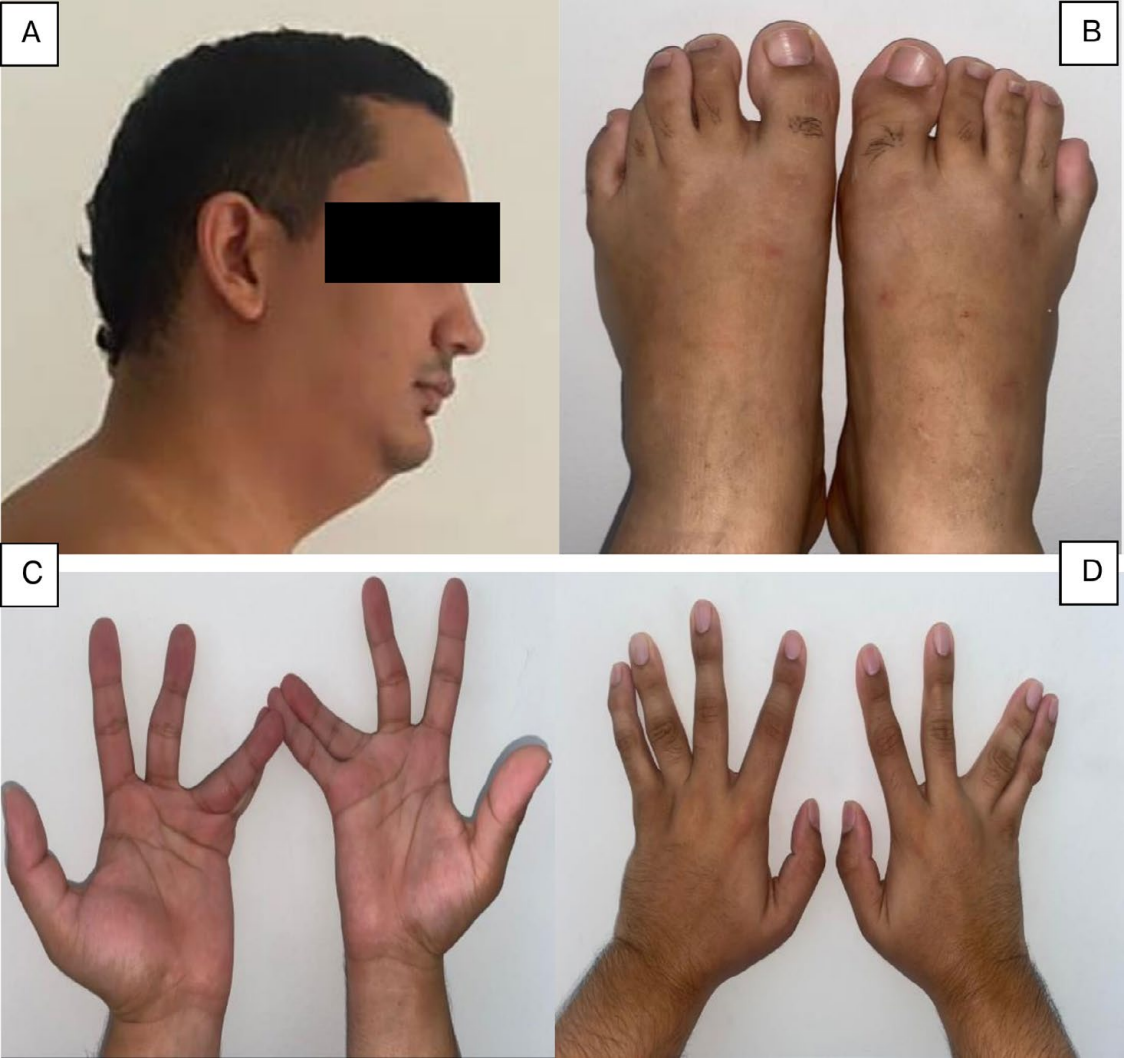
Phenotypic features. (A) Micrognathia and short broad neck. (B) Overlapping of the third toe over the second and fourth toes of the left foot, and overlapping of the third toe over the 2nd toe of the right foot. (C, D) Bilateral ulnar deviation of the fourth and fifth fingers.

At 23 years old, the patient presented to a medical genetics clinic with a history of hypothalamic hamartoma and gelastic seizures, a history of postaxial polydactyly in the hands and feet, and craniofacial dysmorphisms. He weighed 85.3 kg, was 172 cm tall, and had a head circumference of 57.2 cm. On physical examination, the patient exhibited dolichocephaly, bilateral epicanthus, a broad nasal bridge, a short and broad neck, mild cervical kyphosis, mild scoliosis (Figure 1), micrognathia, bilateral ulnar deviation of the 4th and 5th fingers, overlapping of the 3rd toe over the 2nd and 4th toes of the left foot, and overlapping of the 3rd toe over the 2nd toe of the right foot (Figure 2). No genital abnormalities were observed, and neuropsychological evaluations revealed an IQ score using the WAIS‐III scale of 78 (borderline low), although the patient is currently pursuing a university degree.

**FIGURE 3:**
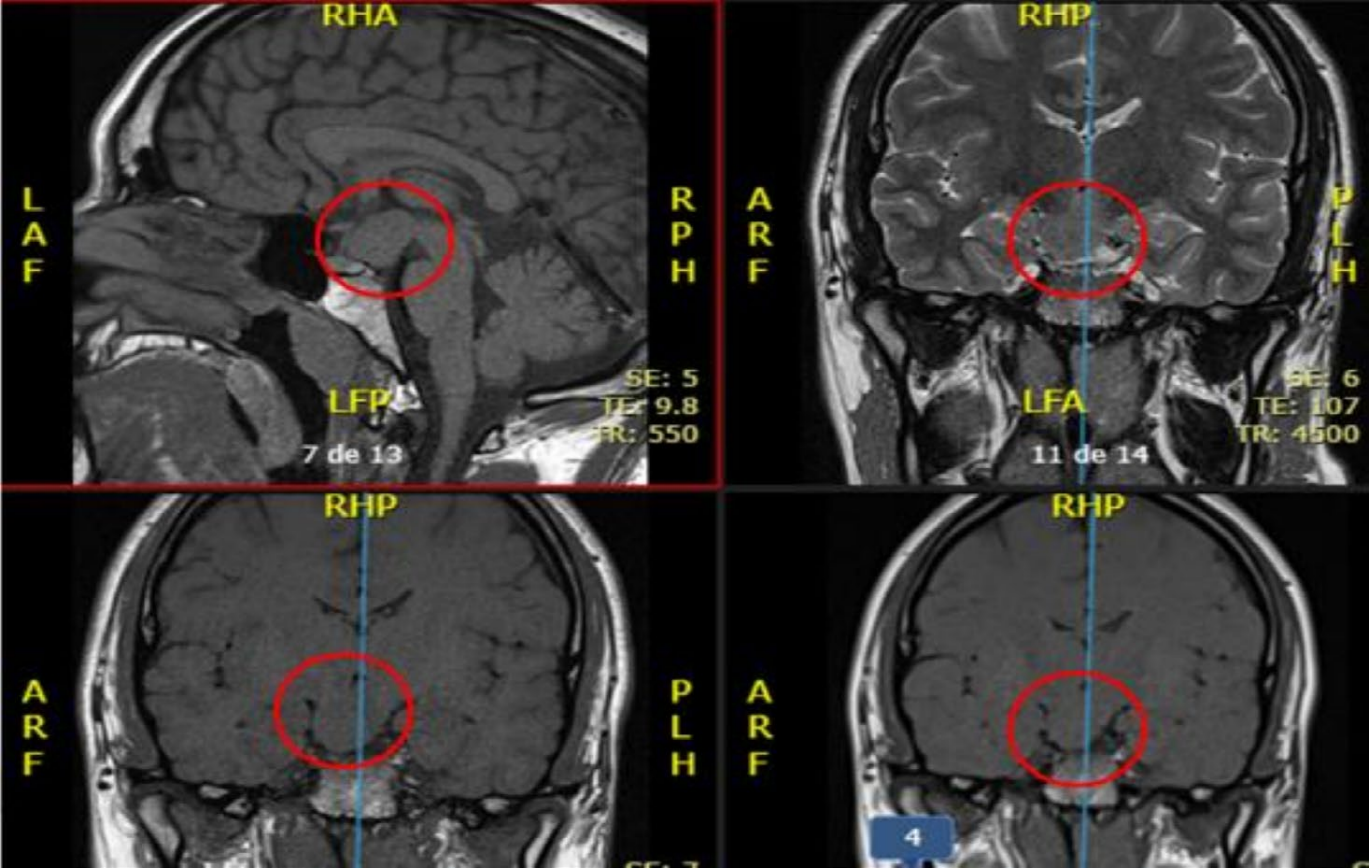
Magnetic resonance imaging (MRI) of the brain with sagittal and coronal views, performed at 22 years of age, showing a hypothalamic hamartoma centered on the tuber cinereum with cisternal and intraventricular involvement (red circle).

**FIGURE 4:**
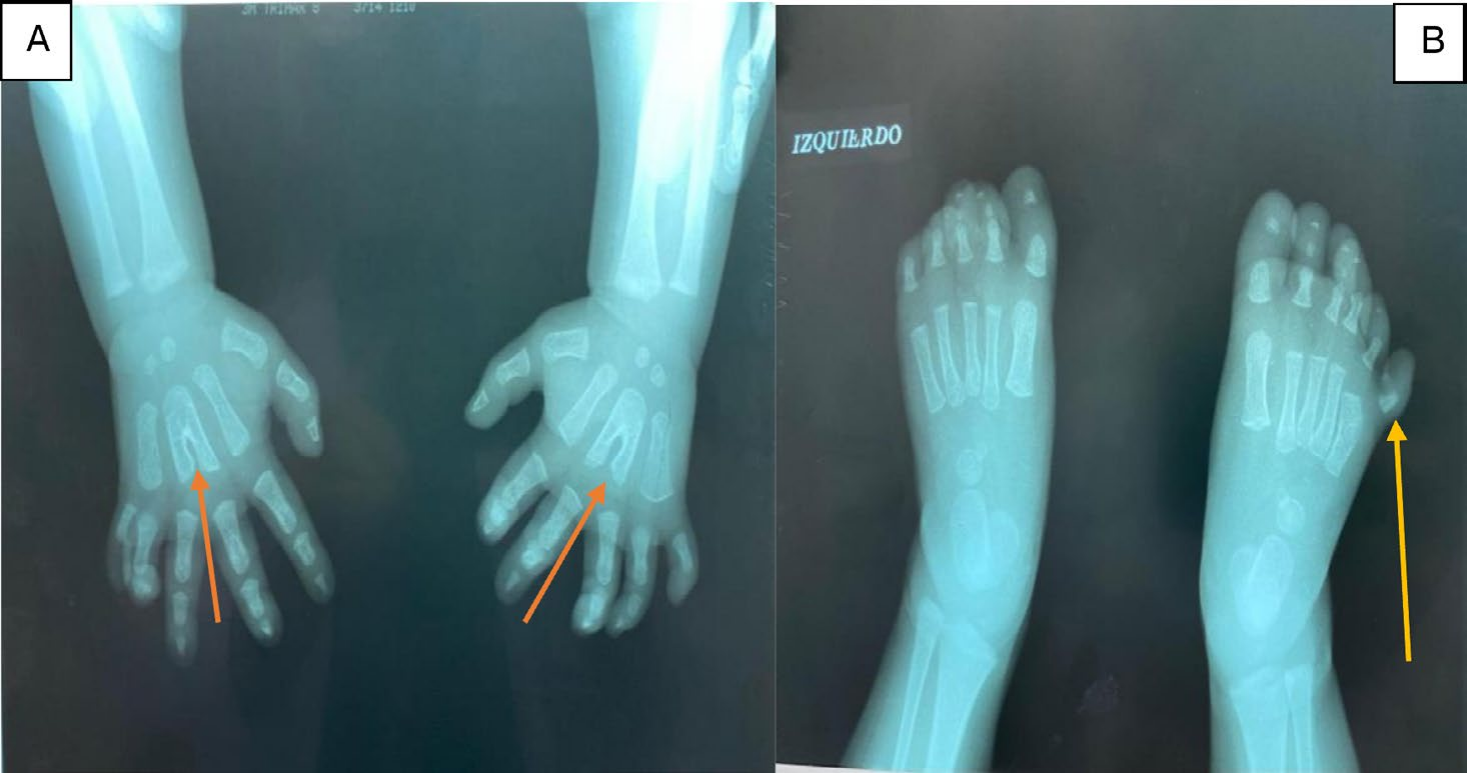
Radiographic images of the hands and feet at 3 months of age, prior to surgery. (A) Hands: Postaxial polydactyly with fusion of the 3rd and 4th metacarpals in a “Y‐shape” in both hands (orange arrow). (B) Feet: Postaxial polydactyly of the right foot (yellow arrow).

**FIGURE 5:**
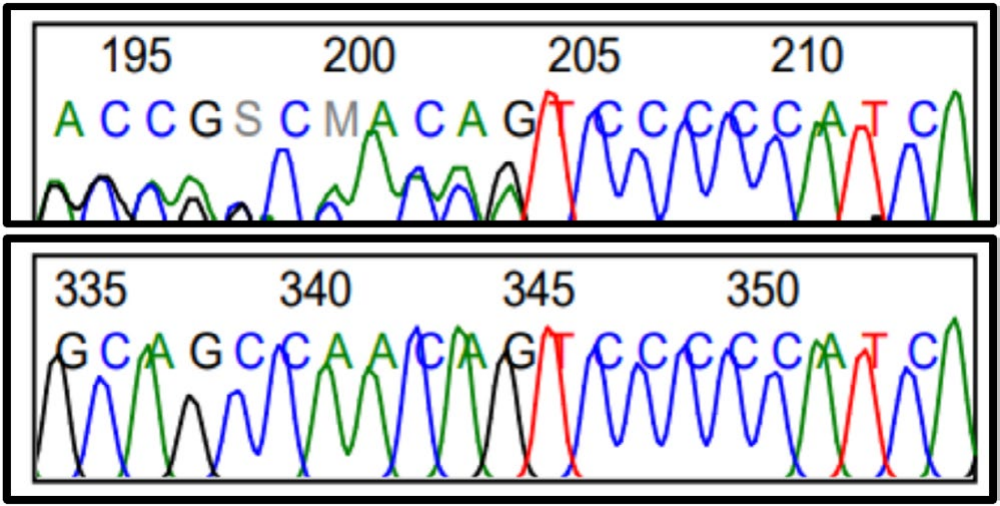
Sanger sequencing showing the *GLI3* variant c.2151del; p.(Gln71HisfsTer16). Reference genome: Hg38.

A G‐banding karyotype from peripheral blood performed in childhood showed a normal chromosomal sex (46, XY). Brain MRI and skull base MRI revealed a hypothalamic hamartoma centered on the tuber cinereum (Figure 3). (In the context of a clear clinical and genetic diagnosis of Pallister‐Hall syndrome, magnetic resonance imaging is sufficient to identify the hypothalamic hamartoma, and there is no clinical justification for obtaining tissue, as the histopathological diagnosis does not alter therapeutic management or prognosis. Performing a biopsy carries a significant risk of both endocrinological and neurological complications). Hormonal studies were normal, and radiographs of the hands and feet confirmed postaxial polydactyly (Figure 4). An abdominal MRI showed mild hepatic steatosis but no renal malformations. Whole‐exome sequencing revealed a de novo heterozygous pathogenic variant in the *GLI3* gene: c.2151del;p.(Gln71HisfsTer16), which was confirmed by Sanger sequencing (Figure 5). It was not possible to perform familial segregation studies in the patient's parents due to geographic limitations that prevented access to genetic testing. However, considering that the gene involved has complete penetrance for the associated clinical phenotypes, it would be expected that the parents would show some clinical manifestations if they carried the variant. Since both parents are completely healthy, it is inferred that the variant identified in the patient is likely a de novo pathogenic variant, which is further supported by the specific type of variant found.

## 3 Materials and Methods

### 3.1 Ethical Compliance

This study was approved by the Ethics Committee of Fundación Valle del Lili, Colombia, and performed in accordance with the Declaration of Helsinki.

Genomic DNA was purified from a peripheral blood sample using the Maxwell RSC Blood DNA Kit. Libraries were prepared using the HubExome Plus Panel‐GeneSGKIT enrichment‐capture kit according to the manufacturer's protocol. Libraries were quantified using the Qubit dsDNA HS assay kit and a Qubit 2.0 fluorometer and diluted according to the protocol requirements. Sequencing by synthesis and bridge amplification was performed using the Illumina NextSeq500/550 platform. This kit allows for the capture of the entire exome and mitochondrial genome. Exome analysis included detection of point mutations, indels, large indels, ALU insertions, and copy number variations (CNVs). A pathogenic (P) heterozygous variant was detected in the *GLI3* gene.

Variant classification was carried out using the criteria from the American College of Medical Genetics and Genomics (ACMG). The variant meets the PVS1 and PM2 criteria.

The variant meets the PVS1 criterion, which refers to a null variant in a gene where loss of function is a known disease‐causing mechanism. The patient has a frameshift variant, which is classified as a loss‐of‐function variant. Additionally, the variant meets the PM2 criterion, which refers to an extremely low frequency in population databases. In this case, it is the only instance reported in the literature with this variant and with clinical concordance.

## 4 Results

A pathogenic, de novo, heterozygous variant in the *GLI3* gene was observed: c.2151del;p.(Gln71HisfsTer16), detected through whole‐exome sequencing and confirmed by Sanger sequencing (Figure 5).

## 5 Discussion

Pallister‐Hall Syndrome (PHS) is a polymalformative monogenic disorder with an autosomal dominant inheritance pattern, resulting from pathogenic heterozygous variants in the *GLI3* gene. This gene encodes a zinc finger transcription factor and is an important mediator of the Sonic Hedgehog (*SHH*) signaling pathways in primary cilia (Green et al. 2023). The syndrome is characterized by distinctive features including hypothalamic hamartoma, postaxial or mesoaxial polydactyly, anal atresia, and bifid epiglottis, typically observed from the neonatal period (Jaiman et al. 2012). However, advancements in molecular diagnostic techniques, such as next‐generation sequencing, have revealed a broad phenotypic variability in patients with pathogenic variants in the *GLI3* gene and PHS phenotype. Depending on the type of variant, milder phenotypic manifestations may be observed, with some individuals surviving into adulthood (PubMed 2024).

Among the phenotypic variability of PHS patients, hypothalamic hamartoma is a prevalent malformation, found in almost all cases. This malformation often leads to gelastic/dacrystic seizures and panhypopituitarism. In the central nervous system, pituitary aplasia, callosal defects, and holoprosencephaly may be present in a minority of cases (Grassa et al. 2022). Associated craniofacial anomalies include cleft palate, bifid epiglottis and larynx, and multiple oral frenula. Limb anomalies described include postaxial or mesoaxial polydactyly or oligodactyly, small nails, cutaneous syndactyly, clinodactyly, and camptodactyly. Renal malformations such as renal agenesis/hypoplasia or ectopic kidneys, imperforate anus, aganglionic megacolon, anomalous pulmonary lobulation, and congenital heart defects, primarily septal defects, are frequently associated internal malformations. Endocrinological manifestations, such as panhypopituitarism and/or adrenal hypoplasia (adrenal insufficiency), not only complicate the early recognition of the syndrome due to their association with hypothalamic hamartoma but also represent significant causes of severity and morbidity. Rapid detection and endocrine intervention for hormonal deficits can improve patient outcomes (Bönnemann et al. 2023).

The degree of overlap, considering the clinical heterogeneity, has been noted in multiple clinical entities, presenting with very similar malformative defects. These include oral‐facial‐digital syndromes (OFDS, particularly type VI), hydroletal syndrome, Smith‐Lemli‐Opitz syndrome type II, some short‐rib polydactyly syndromes, McKusick‐Kaufmann syndrome, Pallister‐Hall‐like syndrome due to allelic variants in the *SMO* gene, trisomy 13, and other ciliopathies. Indeed, hypothalamic masses have been found as part of their clinical spectrum (Verloes et al. 1995).

**FIGURE 6:**
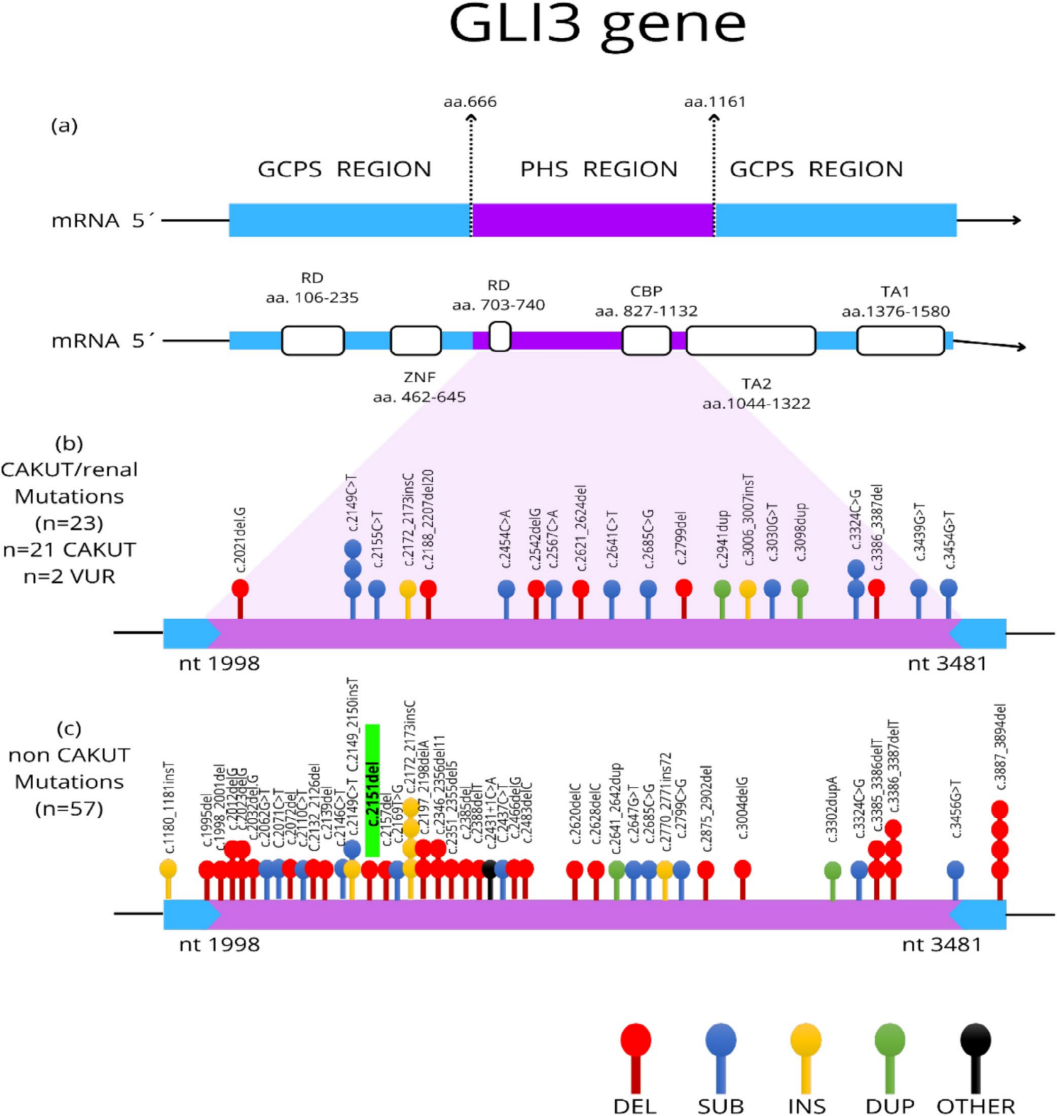
Variant map of the *GLI3* gene. (a) Pallister‐Hall syndrome (PHS) region between amino acids 666 and 1161 of the *GLI3* gene. (b) Variants in 21 patients with congenital anomalies of the kidney and urinary tract (CAKUT) and two patients with vesicoureteral reflux (VUR). (c) Variants in 57 non‐CAKUT patients. Adapted from McClelland et al. (2022).

Regarding the different phenotypes observed within the clinical spectrum of the *GLI3* gene, Greig cephalopolysyndactyly syndrome (GCPS) is noted. This polymalformative syndrome predominantly affects the facial and limb appearance more extensively than PHS. GCPS rarely presents with hypothalamic hamartoma and is associated with pathogenic variants in the *GLI3* gene in regions different from those causing PHS (the distal two‐thirds of the gene) (Figure 6). The phenotypic characteristics of GCPS and other similar clinical entities described earlier may overlap with PHS, posing a diagnostic challenge. Therefore, comprehensive molecular studies, such as whole‐exome sequencing (including germline‐associated genes for hypothalamic hamartoma like *OFD1, CPLANE1, SMO, MAN2C1)* (Green et al. 2023), and the interpretation of variants in relation to their position within the gene, along with knowledge of the clinico‐molecular characterization involving this gene, are essential tools for diagnosis, clinical management, prognosis, treatment, and genetic counseling (McClelland et al. 2022).

Most pathogenic variants associated with Pallister‐Hall Syndrome (PHS) occur in the middle third of the *GLI3* gene (exons 13–15 and amino acids 667 to 1161) (Yang et al. 2021) (Figure 6). To date, more than 70 pathogenic variants of GLI3 have been described in patients with PHS, with most variants being frameshift and nonsense types that lead to loss of protein function (McClelland et al. 2022). Unlike PHS, Greig cephalopolysyndactyly syndrome (GCPS), as part of the *GLI3*‐associated phenotype, is often caused by any type of variant, including missense variants. In this study, we identified the frameshift variant c.2151del;p.(Gln71HisfsTer16) in our patient, adding to the knowledge of pathogenic *GLI3* variants responsible for PHS phenotypes. This study represents the first official case report of a PHS patient in Colombia and one of the few reported cases of PHS in adulthood. Additionally, it describes a new pathogenic variant in the *GLI3* gene that has not been previously reported in the literature or in ClinVar variant databases, although it meets pathogenicity criteria according to ACMG guidelines (criteria PVS1 and PM2). Our group reports this variant in ClinVar as disease‐causing with clear clinical congruence.

Additionally, our case contributes to the existing literature on Pallister‐Hall syndrome (PHS) by providing a more detailed description of the phenotypic characteristics in patients from the Latin American population, which has been scarcely characterized both clinically and molecularly compared to other populations. This case, diagnosed late in adulthood, reinforces previously described genotype–phenotype correlations, as evidenced by the presence of hypothalamic malformation, characteristic radiological findings at the metacarpal level, and the particular pattern of epileptic seizures associated with this syndrome. A review of several patients with PHS was conducted based on their clinical and molecular characteristics (Table 1).

**TABLE 1:** Clinical features and molecular outcome of GLI3 in some cases of PHS.

	cDNA alteration (c.)	Predicted protein alteration (p.)	Inheritance	Growth delay/GHD	Hypothalamic hamartoma	Brachytelephalangism/dactyly	Y‐shaped metacarpal/metatarsal	Insertional/postaxial PD	Craniofacial anomalies	Anal atresia	Bifid epiglottis	Cardiac anomalies	Renal anomalies	Genital anomalies	Lung dysplasia
This case	2151del	Gln717Hisfs*16	De novo	+	+	−	+	+	+	−	−	−	−	−	−
Démurger et al. 2015	2072del	Gln691Argfs*2	De novo	+	+	NI	+	+	−	−	NI	−	−	+	−
Démurger et al. 2015	2149C>T	Gln717*	De novo	+	+	+	+	+	−	+	NI	−	+	+	−
Démurger et al. 2015	2641_2642dup	Gln881Hisfs*10	De novo	+	+	+	+	+	NI	+	NI	+	−	+	NI
Démurger et al. 2015	3040G>T	Glu1014*	De novo	+	+	+	+	+	+	+	−	+	+	+	+
Yang et al. 2021	c.2331C>G	p.His777Gln	De novo	+	+	−	NI	+	NI	NI	+	NI	NI	NI	NI
Démurger et al. 2015	c.1995delT	p.(Gly666Alafs*27)	De novo	−	+	+	+	−	+	+	+	−	−	−	−
Vanderheyden et al. 2024	c.1995delT	p.(Gly666Alafs*27)	De novo	−	+	NI	+	+	NI	NI	NI	NI	+	NI	NI

*Note:* Adapted from Démurger et al. (2015).

Abbreviations: GHD, growth hormone deficiency; NA, Not applicable; NI, no information; PD, polydactyly.

Literature on the clinico‐molecular correlation of pathogenic *GLI3* gene variants shows that frameshift variants due to small deletions are more frequently associated with hypothalamic hamartoma with refractory gelastic seizures, postaxial polydactyly, and metacarpal fusion in a “Y‐shape” (Démurger et al. 2015). Additionally, these variants are more common, though not exclusively, associated with the absence of renal malformations (Figure 6), which are often present in PHS (McClelland et al. 2022). The phenotypic characteristics observed in our patient correlate with the genetic variant present, contributing to the genotype–phenotype characterization in patients with PHS and pathogenic *GLI3* variants (Table 1).

Currently, the patient is receiving comprehensive treatment, including anticonvulsant management with levetiracetam, clobazam, lacosamide and occupational therapies, along with follow‐up by multiple specialties such as neurology, endocrinology, and neuropsychology. The patient's progress and follow‐up are being supervised at the medical genetics clinic, and genetic counseling has been provided to the patient and their family.

## 6 Conclusions

This case expands the current understanding of Pallister‐Hall syndrome by describing the first genetically confirmed patient in Colombia and reporting a novel frameshift pathogenic variant in the *GLI3* gene, c.2151del;p.(Gln71HisfsTer16), which has not been previously documented in variant databases or the literature. The clear genotype–phenotype correlation observed—characterized by hypothalamic hamartoma with refractory gelastic seizures, postaxial polydactyly, and characteristic craniofacial features—reinforces established associations between frameshift variants and the classical PHS phenotype. Moreover, this report underscores the diagnostic value of exome sequencing in adult patients with syndromic features whose diagnosis was delayed beyond childhood. By providing detailed phenotypic and molecular characterization in a Latin American individual, this case contributes to addressing the underrepresentation of such populations in the literature and variant databases; the findings emphasize the relevance of multidisciplinary management, including neurological, endocrinological, and genetic follow‐up, as well as the indispensable role of genetic counseling for patients and at‐risk relatives. This case broadens the clinical spectrum regarding the viability of patients with PHS into adulthood, showing that it is not restricted to the severe neonatal or infantile presentations classically described.

## Author Contributions

All authors listed have made a substantial, direct, and intellectual contribution to the work and approved it for publication.

## Consent

Written informed consent was obtained from the patient for publication of this case report and any accompanying images. A copy of the written consent is available for review by the Editor‐in‐Chief of this journal.

## Conflicts of Interest

The authors declare no conflicts of interest.

## Data Availability

The data that support the findings of this study are available from the corresponding author upon reasonable request.
